# Common carotid artery intima-media thickness increases throughout the pregnancy cycle: a prospective cohort study

**DOI:** 10.1186/s12884-018-1841-y

**Published:** 2018-05-31

**Authors:** Nancy Anderson Niemczyk, Marianne Bertolet, Janet M. Catov, Mansi Desai, Candace K. McClure, James M. Roberts, Akira Sekikawa, Ping Guo Tepper, Emma J. Barinas-Mitchell

**Affiliations:** 1 Department of Epidemiology, Graduate School of Public Health, University of Pittsburgh, 130 De Soto Street, Pittsburgh, PA 15261 USA; 2 Department of Health Promotion and Development, School of Nursing, University of Pittsburgh, 3500 Victoria Street, 440 Victoria Building, Pittsburgh, PA 15261 USA; 3 Department of Obstetrics and Gynecology, School of Medicine, University of Pittsburgh, 3550 Terrace Street, Pittsburgh, PA 15213 USA; 4 Department of Clinical and Translational Research, School of Medicine, University of Pittsburgh, 3550 Terrace Street, Pittsburgh, PA 15213 USA; 5 Magee-Womens Research Institute, Magee-Womens Hospital of University of Pittsburgh Medical Center (UPMC), 204 Craft Avenue, Pittsburgh, PA 15213 USA

**Keywords:** Common carotid artery intima-media thickness, Inter-adventitial diameter, Pregnancy, Cardiovascular disease, Vascular remodeling

## Abstract

**Background:**

High parity is associated with greater cardiovascular disease (CVD) among mid-life and older women. Prospective studies of arterial change throughout pregnancy are needed to provide insight into potential mechanisms. This study assessed vascular adaptation across pregnancy in healthy first-time pregnant women.

**Methods:**

The Maternal Vascular Adaptation to Healthy Pregnancy Study (Pittsburgh, PA, 2010–2015) assessed 37 primigravid women each trimester, 6–8 weeks after delivery and 1–5 years postpartum, with B-mode ultrasound imaging of common carotid artery (CCA) intima-media thickness (IMT) and inter-adventitial diameter (IAD) to assess associations with physical and cardiometabolic measures.

**Results:**

Thirty-seven women (age 28.2 ± 4.5 years, pre-pregnant BMI 24.4 ± 3.2 kg/m^2^) experienced uncomplicated pregnancies. After adjustment for age and pre-pregnancy BMI, mean (SE) IAD (mm) increased each trimester, from 6.38 (0.08) in the 1st trimester to 6.92 (0.09) in the 3rd trimester, and then returned to 1st trimester levels postpartum (6.35 [0.07], *P* <  0.001). In contrast, mean (SE) CCA IMT (mm) increased from the 2nd trimester (i.e., 0.546 [0.01]) onward, and remained higher at an average of 2.7 years postpartum (0.581 [0.02], *P* = 0.03). Weight partially explained changes in IAD.

**Conclusions:**

In uncomplicated first pregnancies, IAD increased and returned to 1st trimester levels postpartum. In contrast, CCA IMT remained increased 2 years postpartum. Maternal weight explained vascular changes better than did metabolic changes. Increased postpartum CCA IMT may persist and contribute to long-term CVD risk.

**Electronic supplementary material:**

The online version of this article (10.1186/s12884-018-1841-y) contains supplementary material, which is available to authorized users.

## Background

High parity is associated with greater cardiovascular disease (CVD) risk in women [1]. Although some of this risk may be due to socio-economic status and lifestyle factors associated with greater parity, acute physiologic changes during pregnancy also may contribute to CVD risk [1–4]. For example, either weight gain or the atherogenic metabolic changes of pregnancy may instigate persistent unhealthy vascular changes [5, 6]. However, studies that could illuminate these relationships have been limited by 1) sample sizes inadequate to detect significant differences in vessel measures [7, 8], 2) failure to collect serial arterial measures [6], 3) use of non-standard techniques to assess the vasculature [5, 9], 4) short follow-up [7, 8], and 5) lack of biomarker collection across the pregnancy cycle [5–10].

Structural arterial changes during pregnancy can be assessed using B-mode ultrasonography of the carotid artery, a well-established, non-invasive, reproducible technique [11]. Abnormal values of two measures of arterial structure—greater intima-media thickness (IMT) and inter-adventitial diameter (IAD) of the common carotid artery (CCA)—are associated with greater CVD risk factor burden [12–14], arterial aging [15], and higher incidence of CVD [13, 16, 17]. The normal changes that occur in the CCA IMT and IAD during and after a healthy pregnancy have not been well established.

The primary objective of our *Maternal Vascular Adaptation to Healthy Pregnancy* (MVP) study was to assess vascular changes in normal first pregnancies, using an adequate sample size, serial measures, a standardized technique to assess vasculature, and including collection of biomarkers. We hypothesized that the vasculature would transiently adapt to the increased blood volume and metabolic requirements of healthy pregnancy, and that these adaptations would be associated with pregnancy weight gain and changes in levels of cardiometabolic factors.

## Methods

### Study design and population

The MVP study prospectively assessed common carotid artery measures in a cohort of healthy primigravid women. Eligible participants recruited from the community were healthy, non-smoking primigravid women, aged ≤40 years, at less than 38 weeks of gestational age. Exclusion criteria were the following: 1) vasoactive medication use; 2) infertility history—defined as either experiencing a period of at least 12 months marked by the inability to achieve pregnancy or using fertility medications to achieve pregnancy; 3) family history of premature coronary artery disease; 4) previous abortion; 5) multiple gestation.

Study visits were scheduled at 12–14, 24–26, and 36–38 weeks of pregnancy, and 6–8 weeks postpartum; all visits were conducted between in 2010 to 2013. After telephone screening for eligibility, women began the study at any one of the pregnancy visits. Each visit involved physical measures (e.g., height and weight) and ultrasound measures of the carotid artery. We calculated that 31 women were needed as participants to generate 80% power to detect a 0.5 SD difference for change in CCA IMT and IAD given an assumed 0.5 correlation among the repeated observations. Because we estimated that 1) 10–20% of women develop a pregnancy complication and 2) our study would experience 25% attrition, we targeted recruitment of 46. The study enrolled 44 women, of whom 43 had multiple visits, and six developed pregnancy complications (one preeclampsia; 3 gestational hypertension; 2 preterm births, one of which had a placental abruption), which left 37 participants with uncomplicated pregnancies and full term births of normal weight newborns in the analytic sample for our analysis.

These participants were invited to return for a follow-up visit 1–5 years after their first postpartum visit. Fourteen had moved out of the region and were unable to participate. Participants (i.e., five women) were excluded if they were pregnant or if they had given birth within the previous 4 months, which generated seventeen potential participants. Of these seventeen, fourteen experienced uncomplicated first pregnancies and were, therefore, included in our analysis. These follow-up visits occurred between 2014 and 2015. Participants signed an informed consent document approved by the University of Pittsburgh, Human Research Protection Office.

### Carotid artery measures

Carotid ultrasounds were performed by a trained research vascular sonographer from the University of Pittsburgh, Ultrasound Research Laboratory (URL). Participants were placed supine, with a right hip wedge for comfort if necessary, and the common carotid artery was scanned bilaterally with high-resolution B-mode ultrasound (ACUSON Cypress System, Malvern, PA.) Digitized images of the common carotid artery were obtained at end diastole, 1 cm proximal to the carotid bulb, and IMT was measured as the distance from the media-adventitial interface to the intima-lumen interface of both the near and far wall of the artery. Approximately 140 measurements of thickness were made for each 1-cm segment, and the mean of each segment was calculated. IMT reported represents the mean value for near and far wall bilaterally. IAD was measured as distance from the adventitial-medial interface of the near arterial wall to the media-adventitial interface of the arterial wall using the same CCA segment. Images were read by one reader, using a computerized, semi-automated reading program system [18]. Reproducibility of carotid measures at the URL was excellent during the time period of the study, with an intraclass correlation coefficient within reader of over 0.91 for CCA IMT and over 0.99 for IAD.

### Demographic, pregnancy history, physical, and laboratory measures

At the initial visit, participants completed a self-administered demographic form. Research staff 1) measured the height of participants using a stadiometer and 2) weighed the participants on a standard balance scale. The mean value of two readings for each measure was recorded. Pre-pregnancy weight was identified preferentially as the pre-pregnancy weight documented in the prenatal record or, if not available, as a documented weight in the medical record in the 3 months prior to the last menstrual period. Pre-pregnancy BMI was calculated as pre-pregnancy weight in kilograms divided by height in meters, squared. Weight change was calculated as the difference between current and pre-pregnancy weight.

Pulse and blood pressure were measured, according to a standardized protocol. Three measurements of each were taken, and the mean of the last two measurements was recorded and used for our analysis. Data resulting from both demographic and physical measures and records reviews were collected and managed using REDCap electronic data capture tools hosted at the University of Pittsburgh [19].

Laboratory assays of fasting serum samples collected at each visit were performed at the Heinz Nutrition Laboratory at the University of Pittsburgh, Graduate School of Public Health, and the following parameters were determined using standard laboratory procedures: total cholesterol, high density lipoprotein (HDL-c), low density lipoprotein (LDL-c) [20], triglycerides [21], and glucose [22]. Insulin was measured using a standard radio-immune assay (Linco Research, St. Charles, MO). HOMA-IR, a measure of insulin resistance, was calculated as (glucose (mg/dl) x insulin (μU/ml))/405 [23]. High-sensitivity C-reactive protein (hsCRP) was measured with an enzyme-linked immunoassay (Alpha Diagnostics International Inc., San Antonio, TX).

Prenatal and birth records were reviewed after the first postpartum study visit to exclude women with complications, which included gestational hypertension, preeclampsia, and preterm birth. Participants completed an interval reproductive and health history form at the second postpartum visit.

### Statistical analysis

Measures with normal distributions were evaluated as means ± standard deviations. Measures with non-normal distributions (i.e., hsCRP and HOMA-IR) were analyzed as medians with interquartile range and log-transformed for our analysis. Categorical variables (e.g., employment) were presented as percentages. Linear mixed models featuring random intercepts and Toeplitz variance and covariance structure were used to estimate means for CCA IMT and IAD.

Baseline maternal age and pre-pregnancy BMI were included a priori in all models. Separate models were constructed for systolic blood pressure, weight, and weight change. For CCA IMT, models were also constructed including IAD, since over time increases in IAD can cause increases in CCA IMT. Predictors with a significance level of *P* ≤ 0.2 were then placed into models together, and predictors with a significance level of *P* ≤ 0.1 were retained. Next, biomarkers were tested individually in the final models identified for each outcome. Biomarkers with a significance level of *P* ≤ 0.1 were then placed into the best models together, and significant predictors were retained. A sensitivity analysis was performed to eliminate three extreme outlier values for hsCRP (i.e. ≥ 60 mg/L). *P* values of 0.05 or less were considered statistically significant for the analysis. As a sensitivity analysis, the analysis was repeated using only data from women who completed all four initial visits. Associations between physical and carotid measures were not assessed for the second postpartum visit because 1) associations may differ during pregnancy as a result of dramatic hematologic and hormonal changes and 2) the sample size was smaller (i.e., 14) for this visit. Statistical analyses were performed using SAS statistical software releases 9.3 and 9.4 (SAS Institute, Cary, NC).

## Results

The mean number of initial study visits was 3.3 (range 2–4), and 15 participants (41%) completed all 4 visits. The average participant age was 28.4 ± 4.6 years, and the average participant pre-pregnancy BMI was 24.3 ± 3.3. Participants were predominantly white (91.9%), married or living as married (89.2%), well-educated (89.1% college graduate or greater), and employed (64.9% full-time; 24.3% part-time). Mean birth weight was 3427.2 ± 224.5 g and mean gestational age at birth was 39.7 ± 1.3 weeks. Route of birth was vaginal for 91.2% of women, and no newborns had apgar scores less than 7 at 1 or 5 min of life. At the 6–8-week postpartum visit, 88% of participants were breastfeeding their infants exclusively. Fourteen participants completed the second postpartum visit 1–5 years (mean 2.7 years) after their first birth, and seven of these participants had experienced subsequent pregnancies (i.e., five participants reported having one additional birth, one participant reported having two additional births, and one participant having a spontaneous abortion).

**Fig. 1:**
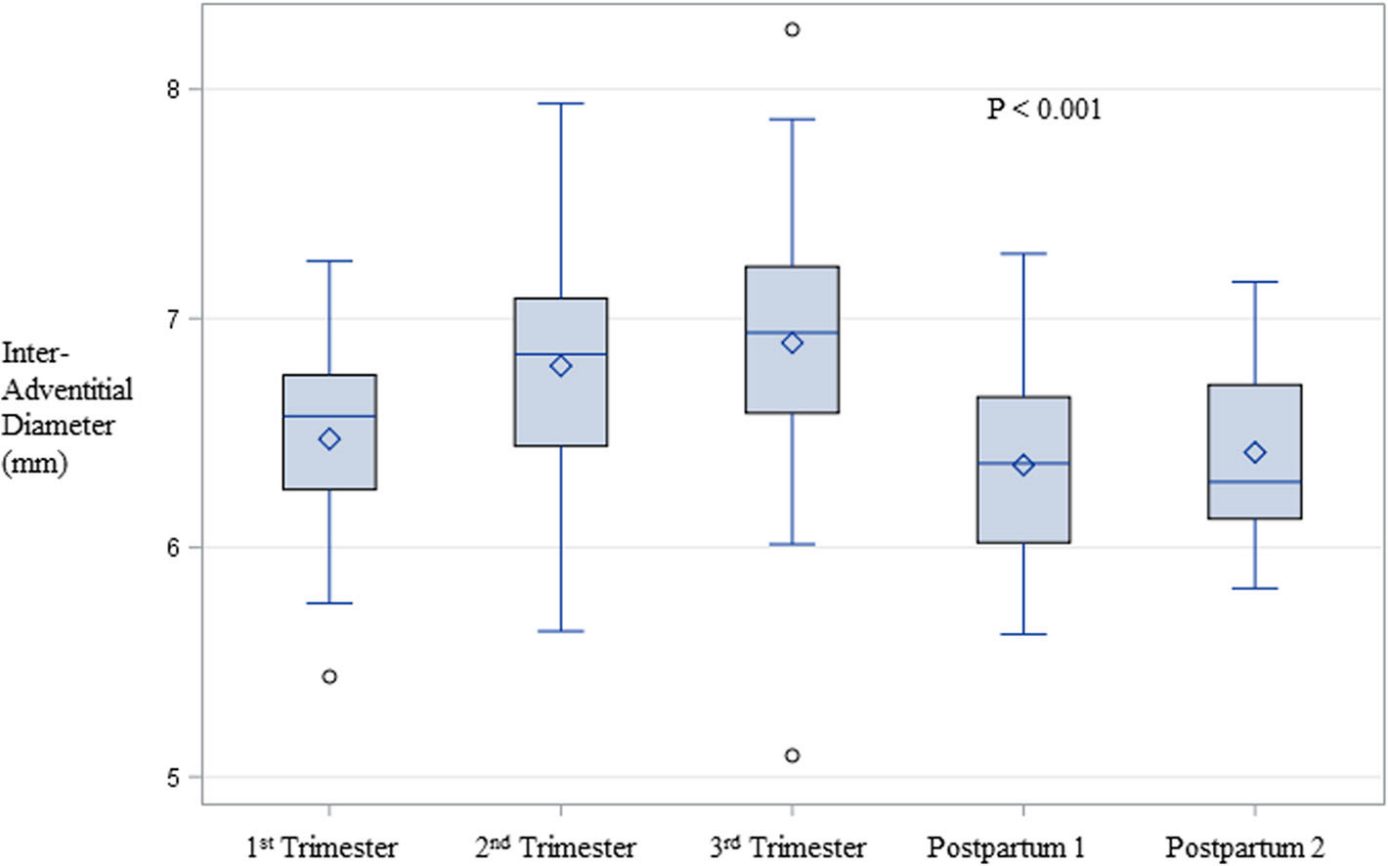
Changes in inter-adventitial diameter across pregnancy, adjusted for maternal age and pre-pregnancy BMI. All pairwise comparisons significant at *P* < .0001 except: 1st Trimester vs. 1st Postpartum *P* = .0.99, 1st Trimester vs. 2nd Postpartum *P* = 0.80, 2nd Trimester vs. 3rd Trimester *P* = .03, 1st postpartum vs. 2nd postpartum *P* = 0.73. Adjusted for age and pre-pregnancy body mass index. The diamond represents the mean and the horizontal line represents the median

**Fig. 2:**
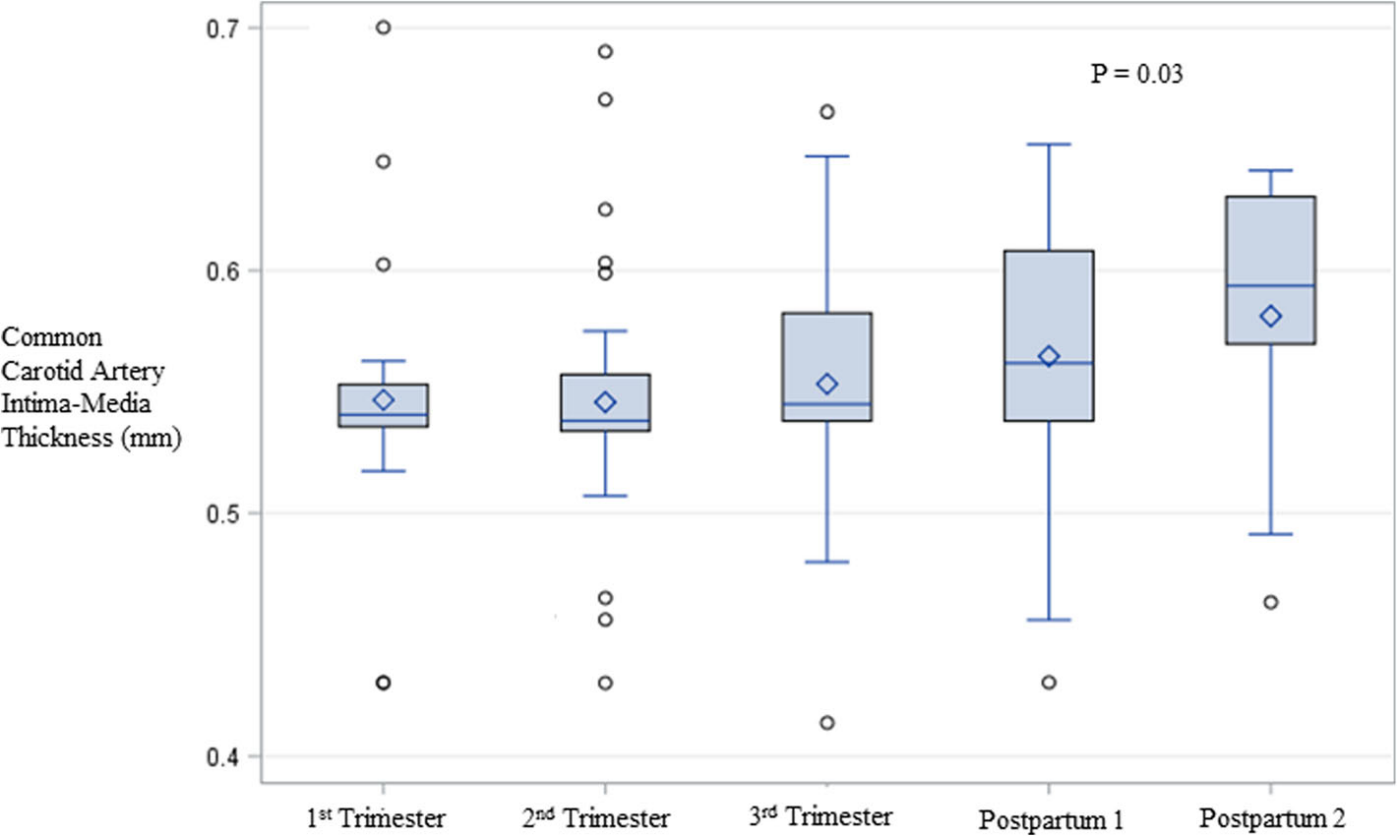
Changes in CCA IMT across pregnancy, adjusted for maternal age and pre-pregnancy BMI. Statistically significant differences are as follows: 1st Trimester vs. 1st Postpartum *P* = 0.03, 1st Trimester vs. 2nd Postpartum *P* = 0.01, 2nd Trimester vs. 1st Postpartum *P* = 0.01, 2nd Trimester vs. 2nd Postpartum *P* = 0.01. The diamond represents the mean and the horizontal line represents the median

Among the participants, IAD increased throughout pregnancy from a mean (SE) of 6.47 (.12) mm in the 1st trimester to 6.89 (.10) mm in the 3rd trimester (all *P* <  0.05). IAD then returned to early pregnancy values (i.e., 6.36 [.07] mm, *P* = 0.76) by the first postpartum visit, and we observed no further decrease at the second postpartum visit (6.42 [0.11] mm) (Table 1). Adjustment for maternal age and pre-pregnancy BMI minimally affected these estimates (Fig. 1). CCA IMT remained stable between the 1st and 2nd trimesters and then increased in the 3rd trimester and through the postpartum period (i.e., 1st trimester mean [SE] 0.547 [.02] mm, first postpartum 0.565 [.01] mm, second postpartum 0.581 [0.02] mm) (Table 1). These values changed minimally when adjusted for maternal age and pre-pregnancy BMI (Fig. 2).

**Table 1:** Unadjusted values for vascular measures and biomarkers by trimester and postpartum

Measure	1st trimester *n* = 17	2nd trimester *n* = 32	3rd trimester *n* = 37	1st postpartum *n* = 35	2nd postpartum *n* = 14	Overall *P*-value
Inter-adventitial diameter (mm)	6.47 (0.12)	6.79 (0.08)	6.89 (0.10)	6.36 (0.07)	6.42 (0.11)	< 0.0001
CCA intima-media thickness (mm)	0.547 (0.02)	0.546 (0.01)	0.553 (0.01)	0.565 (0.01)	0.581 (0.02)	0.03
Weight (kg)	68.7 (2.2)	73.1 (1.6)	79.5 (1.8)	69.2 (1.5)	70.2 (1.9)	< 0.0001
Weight change (kg)	0.55 (0.46)	7.27 (0.62)	14.4 (0.91)	4.3 (0.75)	3.7 (1.1)	< 0.0001
Systolic blood pressure (mm Hg)	103.7 (2.1)	106.0 (1.7)	110.4 (1.4)	106.2 (1.7)	102.9 (2.5)	< 0.0001
Heart rate (bpm)	78.0 (2.4)	79.8 (1.6)	82.0 (1.5)	68.1 (1.5)	63.6 (2.5)	< 0.0001
Total cholesterol (mg/dl)	201.7 (8.9)	257.3 (7.0)	273.1 (7.2)	191.4 (5.5)	194.8 (8.4)	< 0.0001
LDL-c (mg/dl)	111.2 (7.0)	148.0 (6.3)	155.5 (6.6)	114.7 (4.8)	121.9 (7.0)	< 0.0001
Triglycerides (mg/dl)	108.3 (10.5)	176.5 (10.2)	250.7 (13.6)	77.3 (7.0)	87.1 (9.6)	< 0.0001
HDL-c (mg/dl)	68.8 (2.2)	74.0 (3.1)	66.8 (2.3)	61.2 (1.8)	55.6 (2.9)	< 0.0001
Glucose (mg/dl)	79.3 (1.5)	77.2 (1.1)	77.0 (1.2)	82.6 (1.2)	88.5 (1.9)	< 0.0001
Insulin (μU/ml)	8.84 (0.78)	11.25 (0.98)	11.95 (0.81)	8.59 (0.50)	10.76 (0.82)	0.0008
HOMA-IR	1.64 [1.32, 2.09]	2.16 [1.56, 2.56]	2.28 [1.67, 2.51]	1.72 [1.34, 2.13]	2.20 [1.76, 2.87]	0.03
hsCRP (mg/L)	3.58 [2.16, 5.57]	3.36 [2.31, 5.49]	3.29 [2.24, 7.01]	1.20 [.77, 2.44]	0.96 [0.37, 1.50]	< 0.0001

Normally distributed values presented as mean (SE) and *P* value from mixed models. Skewed values presented as median [IQR] and *P*-value from Wilcoxon rank-sum test. CCA is common carotid artery. Weight change is change from pre-pregnancy weight

Changes in weight, blood pressure, heart rate, lipid, glucose, and hsCRP concentrations followed expected patterns for healthy pregnancies [24] (Table 1). Greater weight was associated marginally with greater IAD, and attenuated the increase in IAD that occurred throughout pregnancy (Table 2, Model 3). When metabolic factors were considered, higher triglyceride concentrations were associated (*P* <  0.0001) with lower IAD, but higher hsCRP was associated (*P* = 0.0002) with greater IAD (Table 2, Model 5, and Table 3).

**Table 2:** Associations^a^ between inter-adventitial diameter, physical predictors, and significant metabolic predictors

Predictor	Unadjusted		Model 1^b^		Model 2^b^	
	ß (SE)	*P*-value	ß (SE)	*P*-value	ß (SE)	*P*-value
Trimester 1	Ref		Ref		Ref	
Trimester 2	0.361 (.07)^c^	0.0001	0.361 (.07)^c^	< 0.0001	0.389 (.07)^c^	< 0.001
Trimester 3	0.498 (.07)^cd^	0.0001	0.499 (.07)^cd^	< 0.0001	0.511 (.07)^c^	< 0.001
Postpartum	−0.015 (.05)	0.74	−0.014 (.05)	0.76	0.010 (.04)	0.81
Age (years)			−0.004 (.02)	0.81	−0.003 (.02)	0.83
Pre-pregnancy BMI (kg/m^2^)			0.046 (.02)	0.06	0.042 (.02)	0.09
SBP (mmHG)					0.004 (.00)	0.29
Predictor	Model 3^b^		Model 4^b^		Model 5^b^	
	ß (SE)	*P*-value	ß (SE)	*P*-value	ß (SE)	*P*-value
Trimester 1	Ref		Ref	0.84	Ref	
Trimester 2	0.294 (.09)^c^	0.001	0.321 (.09)^c^	< 0.001	0.456 (.07)^c^	< 0.0001
Trimester 3	0.338 (.13)^c^	0.009	0.392 (.14)^c^	0.008	0.683 (.12)^cd^	< 0.0001
Postpartum	−0.032 (.05)	0.49	−0.020 (.05)	0.69	− 0.029 (.04)	0.44
Age (years)	0.004 (.02)	0.82	− 0.001 (.02)	0.93	−0.006 (.02)	0.73
Pre-pregnancy BMI (kg/m^2^)	0.013 (.03)	0.67	0.047 (.02)	0.06	0.006 (.03)	0.85
Weight (kg)	0.015 (.01)	0.08			0.011 (.01)	0.12
Weight change (kg)			0.010 (.01)	0.29		
Triglycerides (mg/dl)					−0.002 (.00)	< 0.0001
Log hsCRP (mg/L)					0.070 (.02)	0.0002

^a^Linear mixed models

^b^Model 1: Adjusted for age & pre-pregnancy BMI. Model 2: Model 1 plus SBP. Model 3: Model 1 plus weight. Model 4: Model 1 plus weight change. Model 5: Model 3 plus triglycerides and Log hsCRP

^c^Different from postpartum at *p* < .01. ^d^Different from second trimester at *p* < .05

Weight change is from pre-pregnancy weight. β represents change in millimeters

**Table 3:** Associations^a^ of individual biomarkers with inter-adventitial diameter and common carotid artery intima-media thickness

Biomarker	Inter-adventitial Diameter^b^		Common Carotid Artery Intima-Media Thickness^b^	
	β (SE)	*P*-value	β (SE)	*P*-value
Total Cholesterol (mg/dl)	−0.001 (0.1)	0.18	−0.000 (.00)	0.95
HDL-c (mg/dl)	0.002 (.00)	0.52	−0.000 (.00)	0.39
Triglycerides (mg/dl)	−0.001 (.00)	0.01	0.000 (.00)	0.45
LDL-c (mg/dl)	−0.001 (.00)	0.38	−0.000 (.00)	0.80
hsCRP (mg/L)	0.004 (.00)	0.03	0.000 (.00)	0.54
Fasting insulin (μU/ml)	0.004 (.01)	0.55	−0.002 (.00)	0.13
Fasting glucose (mg/dl)	−0.005 (.00)	0.13	−0.001 (.00)	0.09
Log HOMA-IR	−0.013 (.07)	0.86	−0.029 (.01)	0.02

^a^Linear mixed models

^b^Models include time point in pregnancy cycle (trimester or postpartum), age, pre-pregnancy BMI, systolic blood pressure, and weight change from pre-pregnancy baseline

β represents change in millimeters

Higher SBP was associated with greater CCA IMT; nonetheless, accounting for SBP did not attenuate the postpartum increase in CCA IMT (Table 4, Model 2). Greater weight gain was marginally associated with thinner CCA IMT (Table 4, Models 5, 6, 7), and greater IAD was associated with thicker CCA IMT (Table 4, Model 6). In addition, when metabolic factors were considered, greater HOMA-IR was associated with lower CCA IMT values (Table 4, Model 7). Accounting for HOMA-IR did not affect the increased CCA IMT observed postpartum (Table 4, Model 7).

**Table 4:** Associations^a^ between common carotid artery intima-media thickness, physical predictors, and significant metabolic predictors

Predictor	Unadjusted		Model 1^b^		Model 2^b^		Model 3^b^	
	ß (SE)	*P*-value	ß (SE)	*P*-value	ß (SE)	*P*-value	ß (SE)	*P*-value
Trimester 1	Ref		Ref		Ref		Ref	
Trimester 2	0.001 (.01)	0.89	0.001 (.01)	0.89	0.002 (.01)	0.85	0.007 (.01)	0.56
Trimester 3	0.013 (.01)	0.24	0.013 (.01)	0.24	0.009 (.01)	0.43	0.022 (.02)	0.22
Postpartum	0.027 (.01)^c^	0.02	0.027 (.01) ^c^	0.02	0.026 (.01)^cd^	0.03	0.031 (.01)^c^	0.01
Age (yr)			0.004 (.00)	0.03	0.004 (.00)	0.02	0.003 (.00)	0.08
Pre-pregnancy BMI (kg/m^2^)			−0.001 (.00)	0.78	−0.002 (.00)	0.45	0.000 (.00)	0.93
SBP (mm Hg)					0.001 (.00)	0.08		
Weight (kg)							−0.000 (.00)	0.66
Predictor	Model 4^b^		Model 5 ^b^		Model 6^b^		Model 7^b^	
	ß (SE)	*P*-value	ß (SE)	*P*-value	ß (SE)	*P*-value	ß (SE)	*P*-value
Trimester 1	Ref		Ref		Ref		Ref	
Trimester 2	0.016 (.01)	0.19	0.018 (.01)	0.17	0.007 (.01)	0.59	0.009 (.01)	0.51
Trimester 3	0.042 (.02)^c^	0.04	0.041 (.02)^c^	0.046	0.027 (.02)	0.19	0.033 (.02)^c^	0.13
Postpartum	0.036 (.01)^d^	0.003	0.035 (.01)^d^	0.005	0.034 (.01)^c^	0.006	0.027 (.01)^d^	0.03
Age (years)	0.002 (.00)	0.11	0.003 (.00)	0.07	0.003 (.002)	0.05	0.003 (.00)	0.12
Pre-pregnancy BMI (kg/m^2^)	−0.001 (.00)	0.70	−0.002 (.00)	0.35	−0.004 (.00)	0.15	−0.002 (.00)	0.55
Weight change (kg)	−0.002 (.00)	0.13	−0.002 (.00)	0.07	−0.002 (.00)	0.06	−0.002 (.00)	0.08
SBP (mmHg)			0.001 (.00)	0.04	0.001 (.00)	0.04	0.001 (.00)	0.03
Inter-adventitial diameter					0.026 (.01)	0.02	0.017 (.01)	0.17
Log HOMA-IR							−0.028 (.01)	0.03

^a^Linear mixed models

^b^Model 1: Adjusted for age & pre-pregnancy BMI. Model 2: Model 1 plus SBP. Model 3: Model 1 plus weight. Model 4: Model 1 plus weight change. Model 5: Model 1 plus SBP and weight change. Model 6: Model 5 plus inter-adventitial diameter. Model 7: Model 6 plus HOMA-IR

^c^Different from second trimester at *p* < .05. ^d^Different from third trimester at *p* < .05

BMI is body mass index. SBP is systolic blood pressure. Weight change is from pre-pregnancy weight. β represents change in millimeters

Results of sensitivity analyses limited to women who completed all four initial visits and that excluded hsCRP outliers, were consistent with those from the primary analyses (Additional file 1: Table S1 and Additional file 2: Table S2). Moreover, for the second postpartum visit, no reproductive factors (e.g., number of interval pregnancies or breastfeeding status) were statistically significantly associated with either carotid measure (data not shown).

## Discussion

Among our participants with normal first pregnancies, CCA IMT thickened late in pregnancy and remained thickened at 2.7 years postpartum; IAD, however, increased throughout pregnancy and returned to early pregnancy levels, postpartum. Although our results mirror those described in two classic studies [7, 8], our study is the first to follow women for more than 1 year postpartum. With more participants (i.e., 43) than those studies [7, 8] combined, our study establishes statistically significant changes in CCA IMT and IAD. While a recent study did not demonstrate that CCA IMT was increased in the 3rd trimester, it assessed women earlier in the trimester than we did [10]. Our results demonstrate that unhealthy change in CCA IMT is partially explained by changes in IAD and weight—not atherogenic metabolic changes.

An increase in CCA IMT beginning late in pregnancy and persisting postpartum beyond 2 years, in addition to lifestyle changes involved with parenthood and socio-economic profile of women with large families, could help explain the greater CVD risk that occurs for women of high parity [1, 3]. Greater IMT is a risk factor for CVD because thickened arteries are 1) less capable of responding to changes in blood pressure [25] and 2) more prone to atherosclerosis [26]. Although studies have identified greater CCA IMT in women of higher parity [6, 27–29], the cause remains unknown. However, we observed thicker CCA IMT among our participants more than 2 years after childbirth, which suggests that the acute negative effect of pregnancy on CCA IMT may persist and could serve as a risk factor for CVD.

The observed changes in CCA IMT and IAD are consistent with the literature concerning hemodynamic changes in pregnancy and the effect of hemodynamic changes on arteries [30–34]. Importantly, we provide serial measures in pregnancy to characterize this vascular remodeling and evaluate concomitant metabolic markers. Vascular remodeling is largely due to hemodynamic factors. Arterial walls adapt to maintain homeostasis between the two main stresses of blood flow: shear and tensile stress. First, shear stress is the frictional force of blood flowing along the arterial wall. Increased shear stress causes blood vessels to increase in diameter [30–32]. Cardiac output increases early in the 1st trimester of pregnancy [33] and peaks at 30–60% above the non-pregnant level in the late 2nd or early 3rd trimester [33]. Increased cardiac output should increase IAD resulting from increased shear stress, as our results demonstrate. Second, tensile stress is the force of blood perpendicular to the arterial wall, and this force increases as arterial diameter increases, which causes arterial walls to thicken [30, 34]. CCA IMT would thicken during pregnancy as IAD increases, to normalize arterial wall stresses, as our results confirm [25].

In contrast to the effects of body weight and change in IAD, the metabolic changes during pregnancy that may be considered atherogenic in non-pregnant adults (i.e., increased total cholesterol, LDL-c, triglycerides, HOMA-IR, and hsCRP) do not explain the increased IAD and CCA IMT that we observed. As expected, we observed an association between higher hsCRP and greater IAD. Without pregnancy, higher hsCRP concentrations are associated with greater carotid IMT [35–37], which is associated with greater IAD. However, in our study, hsCRP concentrations did not explain the observed changes in IAD. Our finding that higher triglyceride concentrations were associated with smaller IAD [12] was unexpected, because this relationship differs from that observed in non-pregnant adult women.

Triglyceride concentrations increase dramatically during healthy pregnancy to support fetal growth, and no accepted threshold value exists for what constitutes high triglyceride concentrations in pregnancy [24]. However, triglyceride concentrations can be excessive in pregnancy, as triglyceride concentrations in the upper percentiles have been associated with preeclampsia and preterm birth [38–40]. Both high triglyceride concentrations and smaller IAD indeed could be associated with less healthy pregnancies. Our results suggest that paradigms of CVD prediction may not be applicable to the wellness state of pregnancy.

Our study benefited from the use of a highly valid and reproducible measure of carotid structure (i.e., B-mode ultrasonography), and high participant retention (i.e., 98%) in the initial study. We also collected serial vascular and biomarker measures during and after pregnancy, which strengthens this study, but the lack of pre-pregnancy measures poses a limitation. Limitations of the study are largely due to the rapidly changing hormonal and hemodynamic milieus of pregnancy and the postpartum period. Because the hemodynamic changes of pregnancy begin as early as 5 weeks of gestation [33], our 1st trimester values may not represent a true pre-pregnancy baseline. For example, thinning of the CCA IMT may have occurred before we could assess it. Similarly, because most participants (94%) were breastfeeding at the first postpartum visit, their hormonal and cardiovascular status had not attained new postpartum “normal” status. CCA IMT may regress after weaning. Our results also might not reflect those for women who formula-feed. Additionally, at the second postpartum visit, participants exhibited a varying number of subsequent pregnancies, which makes interpretation difficult. However, our results are consistent with those of the Cardiovascular Risk in Young Finns study, which found that young women who gave birth over a 6-year period had greater progression of CCA IMT than those who had not [6], and with epidemiologic studies showing greater CCA IMT in midlife women of higher parity [27–29]. Moreover, although our largely white, well-educated participants do not represent all first-time pregnant women in the United States, our study provides valuable baseline data against which arterial remodeling in other demographic groups can be assessed.

Future work should follow a life course approach, and seek to enroll women during the preconception period to obtain a true baseline and then follow them through at least a several month period after weaning. Retention for the postpartum visits is critical. Additional studies should explore vascular adaptation to pregnancy in women in subsequent pregnancies, from different racial and ethnic groups, and with higher BMI. Collection of serum folate levels might provide valuable insights into the role folate deficiency during pregnancy plays in differences in vascular adaptation.

## Conclusions

We found that IAD increased throughout a healthy first pregnancy and decreased by 8 weeks postpartum. In contrast, postpartum CCA IMT thickening persisted for more than 2 years. These adaptations can be explained—partially—by pregnancy-related changes in weight and IAD; moreover, they are not substantially explained by changes in metabolic measures. Therefore, our results suggest that pregnancy represents a unique setting of rapid physiologic changes that maintain homeostasis during a period of acute stress.

Understanding normal vascular adaptation to pregnancy can not only engender an improved understanding of the physiology of pregnancy complications, but also better identify women at risk for complications early in pregnancy. If it persists, the greater CCA IMT detected postpartum may help explain the higher CVD risk in women of higher parity.

## Additional files

Additional file 1: **Table S1** Associations between inter-adventitial diameter, physical predictors, and significant metabolic predictors for the 15 women who completed all 4 study visits. These are data about carotid measures, physical predictors, and significant metabolic predictors for the 15 women who completed all 4 initial study visits. (DOCX 15 kb)

Additional file 2: **Table S2** Associations between common carotid artery intima-media thickness, physical predictors, and significant metabolic predictors for the 15 women who completed all 4 study visits. These are data about carotid measures, physical predictors, and significant metabolic predictors for the 15 women who completed all 4 initial study visits. (DOCX 15 kb)

## Abbreviations

BMI: Body mass index
CCA IMT: Common carotid artery intima-media thickness
CVD: Cardiovascular disease
HDL-c: High density lipoprotein concentration
hsCRP: High-sensitivity C-reactive protein
IAD: Inter-adventitial diameter
LDL-c: Low density lipoprotein concentration
MVP: Maternal Vascular Adaptations to Healthy Pregnancy
SBP: Systolic blood pressure
SE: Standard error
URL: Ultrasound Research Laboratory
