# Supplementary material for: Common carotid artery intima-media thickness increases throughout the pregnancy cycle: a prospective cohort study
Source: BMC Pregnancy Childbirth. 2018 May 31;18:195. doi: 10.1186/s12884-018-1841-y (PMC5984334; doi:10.1186/s12884-018-1841-y)
Supplement: Supplementary file 1 — Table S1 Associations between inter-adventitial diameter, physical predictors, and significant metabolic predictors for the 15 women who completed all 4 study visits. These are data about carotid measures, physical predictors, and significant metabolic predictors for the 15 women who completed all 4 initial study visits. (DOCX 15 kb) [file 12884_2018_1841_MOESM1_ESM.docx]

**Table S1**

Associations^a^ between inter-adventitial diameter, physical predictors, and significant metabolic predictors for the 15 women who completed all 4 study visits.

| Predictor | Unadjusted | | Model 1^b^ | | Model 2 ^b^ | |
| --- | --- | --- | --- | --- | --- | --- |
|  | ß (SE) | *P*-value | ß (SE) | *P*-value | ß (SE) | *P*-value |
| Trimester 1 | Ref |  | Ref |  | Ref |  |
| Trimester 2 | 0.415 (.08)^c^ | <0.0001 | 0.415 (.08)^c^ | <0.0001 | 0.523 (.10)^c^ | <0.0001 |
| Trimester 3 | 0.566 (.09)^c^ | <0.0001 | 0.566 (.09)^c^ | <0.0001 | 0.684 (.18)^c^ | 0.0005 |
| Postpartum | -0.023 (.05) | 0.63 | -0.023 (.05) | 0.63 | -0.027 (.04) | 0.55 |
| Age (years) |  |  | -0.002 (.03) | 0.96 | 0.007 (.03) | 0.83 |
| Pre-pregnancy BMI (kg/m^2^) |  |  | -0.032 (.06) | 0.59 | -0.082 (.06) | 0.20 |
| Weight (kg) |  |  |  |  | 0.014 (.01) | 0.16 |
| Triglycerides (mg/dl) |  |  |  |  | -0.002 (.00) | 0.0008 |
| Log hsCRP (mg/L) |  |  |  |  | 0.092 (.02) | 0.0001 |

^a^ Linear mixed models

^b^ Model 1: Adjusted for age & pre-pregnancy BMI. Model 2: Model 1 plus age and pre-pregnancy BMI. Model 3: Model 1 plus weight, triglycerides, and Log hsCRP.

^c^Different from postpartum at p <.01.

BMI is body mass index. β represents change in millimeters.
