# Supplementary material for: Common carotid artery intima-media thickness increases throughout the pregnancy cycle: a prospective cohort study
Source: BMC Pregnancy Childbirth. 2018 May 31;18:195. doi: 10.1186/s12884-018-1841-y (PMC5984334; doi:10.1186/s12884-018-1841-y)
Supplement: Supplementary file 2 — Table S2 Associations between common carotid artery intima-media thickness, physical predictors, and significant metabolic predictors for the 15 women who completed all 4 study visits. These are data about carotid measures, physical predictors, and significant metabolic predictors for the 15 women who completed all 4 initial study visits. (DOCX 15 kb) [file 12884_2018_1841_MOESM2_ESM.docx]

**Table S2**

Associations^a^ between common carotid artery intima-media thickness, physical predictors, and significant metabolic predictors for the 15 women who completed all 4 study visits

| Predictor | Unadjusted |  | Model 1^b^ | | Model 2 ^b^ | |
| --- | --- | --- | --- | --- | --- | --- |
|  | ß (SE) | *P*-value | ß (SE) | *P*-value | ß (SE) | *P*-value |
| Trimester 1 | Ref |  | Ref |  | Ref |  |
| Trimester 2 | 0.004 (.01) | 0.75 | 0.004 (.01) | 0.75 | 0.031 (.02)^d^ | 0.11 |
| Trimester 3 | 0.026 (.01) | 0.07 | 0.026 (.01) | 0.07 | 0.075 (.03) | 0.02 |
| Postpartum | 0.038 (.01)^c^ | 0.01 | 0.038 (.01)^c^ | 0.01 | 0.046 (.02) | 0.01 |
| Age (yr) |  |  | 0.002 (.00) | 0.26 | <-0.001 (.00) | 0.98 |
| Pre-pregnancy BMI (kg/m^2^) |  |  | <-0.001 (.00) | 0.94 | 0.002 (.00) | 0.56 |
| Weight change (kg) |  |  |  |  | -0.004 (.00) | 0.09 |
| SBP (mmHg) |  |  |  |  | 0.001 (.00) | 0.21 |
| Log HOMA-IR |  |  |  |  | -0.028 (.02) | 0.10 |
| ^a^ Linear mixed models  ^b^ Model 1: Adjusted for age & pre-pregnancy BMI. Model 2: Model 1 plus weight change, SBP, and Log HOMA-IR.  ^c^Different from second trimester at p <.05. ^d^ Different from third trimester at p <.05.  BMI is body mass index. SBP is systolic blood pressure. Weight change is from pre-pregnancy weight. β represents change in millimeters. | | | | | | |
